# Signal Transduction Pathways (MAPKs, NF-κB, and C/EBP) Regulating COX-2 Expression in Nasal Fibroblasts from Asthma Patients with Aspirin Intolerance

**DOI:** 10.1371/journal.pone.0051281

**Published:** 2012-12-11

**Authors:** Francesc Josep Garcia-Garcia, Joaquim Mullol, Maria Perez-Gonzalez, Laura Pujols, Isam Alobid, Jordi Roca-Ferrer, Cesar Picado

**Affiliations:** 1 Immunoal·lèrgia Respiratòria Clínica i Experimental (IRCE), Institut d’Investigacions Biomèdiques August Pi i Sunyer (IDIBAPS), Barcelona, Catalonia, Spain; 2 Centro de Investigaciones Biomédicas en Red de Enfermedades Respiratorias (CIBERES), Barcelona, Catalonia, Spain; 3 Unitat de Rinologia i Clínica de l’Olfacte, Servei d’Otorinolaringologia, Hospital Clínic, Barcelona, Catalonia, Spain; 4 Servei de Pneumologia i Al·lèrgia Respiratòria, Hospital Clinic, Universitat de Barcelona, Barcelona, Catalonia, Spain; University of Rochester Medical Center, United States of America

## Abstract

**Background:**

Recent studies have revealed that cyclooxygenase-2 (COX-2) expression is down-regulated in aspirin-induced asthma (AIA). Various signal pathways (MAPKs, NF-κB and C/EBP) are involved in COX-2 regulation.

**Objective:**

To investigate the regulation of COX-2 expression through MAP-kinase pathway activation and nuclear factor translocation in aspirin-induced asthma (AIA).

**Methods:**

Fibroblasts were isolated from specimens of nasal mucosa (NM, N = 5) and nasal polyps (NP, N = 5). After IL-1β (1 ng/ml) incubation, COX-2 and phosphorylated forms of ERK, JNK and p38 MAPK were measured by Western blot. MAPK’s role in IL-1β-induced COX-2 expression was assessed by treating cells with ERK (PD98059), JNK (SP600125) and p38 MAPK (SB203580) inhibitors (0.1–10 µM) prior to IL-1β exposure. NF-κB and C/EBP nuclear translocation was measured by Western blot and TransAM® after IL-1β (10 ng/ml) exposure.

**Results:**

No differences were observed in the MAPK phosphorylation time-course between NM and NP-AIA fibroblasts. The p38 MAPK inhibitor at 10 µM significantly reduced IL-1β-induced COX-2 expression in NM fibroblasts (85%). In NP-AIA fibroblasts the COX-2 inhibition (65%) at 1 and 10 µM was not statistically significant compared to non-treated cells. ERK and JNK inhibitors had no significant effect in either the NM or NP-AIA cultures. The effect of IL-1β on NF-κB and C/EBP subunits’ nuclear translocation was similar between NM and NP-AIA fibroblasts.

**Conclusions:**

These results suggest that p38 MAPK is the only MAPK involved in IL-1β-induced COX-2 expression. NM and NP-AIA fibroblasts have similar MAPK phosphorylation dynamics and nuclear factor translocation (NF-κB and C/EBP). COX-2 downregulation observed in AIA patients appears not to be caused by differences in MAPK dynamics or transcription factor translocation.

## Introduction

Aspirin-induced asthma (AIA) is a syndrome clinically characterized by chronic rhinosinusitis with nasal polyposis (CRSwNP), asthma and bronchoconstriction episodes triggered by the intake of non-steroidal-anti-inflammatory drugs (NSAIDs) [1]. A close relationship has been demonstrated between CRSwNP and AIA, since the prevalence of CRSwNP in AIA may be as high as 60–70%, while in the population of aspirin-tolerant asthmatics it is less than 10% [2].

The pathogenesis of AIA remains poorly understood but accumulated evidence suggests that abnormalities in arachidonic acid metabolism may play a role [2], [3]. Both an overactive 5-lipoxygenase pathway (5-LO) and reduced COX expression have been demonstrated, resulting in increased cysteinyl leukotriene production and reduced PGE_2_ release in AIA [1], [4]–[7]. There are two well-characterized COX enzymes: COX-1, considered a constitutive form involved in cell homeostasis [8], and COX-2, an inducible form activated by pro-inflammatory mediators, growth factors and cytokines. These alterations in AIA patients seem to be present in both the lower [6] and upper airways [4]. In fact, previous studies have reported COX-2 down-regulation in airway fibroblasts obtained from AIA patients [7]. In contrast with asthma, increased COX-2 expression has been reported in other airway inflammatory diseases such as cystic fibrosis [9] and chronic obstructive pulmonary disease [10].

The mechanisms responsible for the reported alterations in the regulation of COX-2 in inflamed NP tissue remain to be clarified. It is well known that inflammatory stimuli elicit cellular responses through the activation of mitogen-activated protein kinases (MAPKs) by phosphorylation. MAPKs regulate various cellular activities, including gene expression, mitosis and programmed death. MAPK-catalyzed phosphorylation functions as a switch for turning the activity of their target proteins on/off [11], [12].

In pluricellular organisms, there are three well-characterized subfamilies of MAPKs: extracellular-signal-regulated kinases, p42/44 (ERK1/2), c-jun amino terminal kinases (JNKs) and p38 MAPKs [11], [12]. So far, it has been demonstrated that MAPK family members play a role in COX-2 gene expression in various cell types, such as HUVECs [13], airway smooth muscle cells [14] and chondrocytes [15]. However, the role of the various MAPKs regulating COX-2 in AIA has never been studied.

COX-2 gene expression is also regulated by the action of several transcription factors, such as NF-κB [16]–[18] and C/EBP [19]. It has been widely demonstrated that NF-κB regulates cell survival and inflammatory responses by acting, at least in part, on the two active κB binding sites described in the COX-2 promoter gene [16], [20]–[22]. Active NF-κB complexes are dimers of combinations of Rel family polypeptides (p50, p52 and p65) that respond to a wide variety of stimuli. The composition of NF-κB dimmers partially determines their biological effects by conditioning nuclear translocation and binding to the κB-regulatory elements [16], [20].

There is also a C/EBP binding site on the human COX-2 promoter, which is involved in COX-2 induction. The three main members of the C/EBP family are C/EBPα, C/EBPβ and C/EBPγ. Their nuclear translocation is induced by pro-inflammatory stimuli, but although all C/EBPs subunits recognize the same DNA sequence, the balance between them and the cell type will determine the activation or repression of the gene expression [23]–[25]. However, the regulation of NF-κB and C/EBP transcription factors in fibroblasts from AIA patients has not been studied.

We hypothesized that the COX-2 down-regulation observed in AIA patients is caused by alterations in the mechanisms regulating the activation of MAPKs and nuclear factor translocation (NF-κB, and C/EBPs). As we have demonstrated in previous studies that the anomalies in the regulation of COX-2 are present in cultured fibroblasts obtained from the NP of AIA patients, the objective of our study was to examine the activation of MAPKs and nuclear factor translocation (NF-κB, and C/EBPs) in fibroblasts derived from nasal mucosa, and from the NP of AIA patients.

## Materials and Methods

### Study Population

NM specimens were obtained from the lower turbinate of 5 non-asthmatic subjects with either septal deviation, turbinate hypertrophy or both who were undergoing nasal corrective surgery. All control subjects had taken aspirin or NSAIDs at clinical dosage without any untoward reactions (asthma and/or rhinitis, urticaria, angioedema or anaphylaxis). NP specimens were collected from patients with aspirin intolerance (NP-AIA) who had undergone endoscopic sinus surgery. The clinical and demographic characteristics of the subjects are shown in **Table 1**. The diagnosis of aspirin intolerance was confirmed by lysine-aspirin nasal challenge, as previously described [26]. None of the control subjects had received oral or intranasal corticosteroid treatment for at least one month before surgery. None of the control subjects or patients had any upper airway infection in the 2 weeks before surgery.

**Table 1:** Epidemiological characteristics of control subjects and patients with NP and AIA.

Characteristics	NM fibroblasts	NP-AIA fibroblasts
**Fibroblast cultures, N**	5	5
**Age, years (mean ± SD)**	55.6±14.5	59.6±11.9
**Females, N (%)**	3 (60)	4 (80)
**Asthma, N (%)**	0 (0)	5 (100)
**Aspirin intolerance, N (%)**	0 (0)	5 (100)
**Atopy, N (%)**	0 (0)	2 (40)
**Intranasal corticosteroid, N (%)**	0 (0)	3 (60)

SD, standard deviation.

### Ethical Declaration

All patients gave their written informed consent to participate in the study, which was approved by the Scientific and Ethics Committee (Comité Étic d'Investigació Clínica) of our Institution (Hospital Clínic de Barcelona).

### Tissue Handling and Cell Culture

NM and NP tissues were cut into 3×3 mm fragments and placed in six-well plates (NUNC, Wiesbaden, Germany) containing Dulbecco's modified Eagle's media (DMEM) supplemented by 10% fetal bovine serum (FBS), 100 IU/ml penicillin, 100 µg/ml streptomycin (Invitrogen, Carlsbad, California, USA) and 2 µg/ml amphotericin B (Sigma, St Louis, MO, USA). Cultures were placed in a 5% CO_2_ humidified incubator at 37°C and the culture media were changed every 2 days. Once the fibroblasts had grown, tissue fragments were removed and the first passage was performed by adding 0.05% trypsin/0.02% ethylenediaminetetraacetic acid (Invitrogen, Carlsbad, California, USA) for 5 min. The reaction was stopped with 10% FBS-supplemented DMEM. Cells were then centrifuged (400 g, 5 min) and seeded in 150 cm^2^ flasks (NUNC). At passages 5 to 6, fibroblasts were cultured in CultureSlides® and flasks to perform culture characterization and experimental protocols. The same batch of FBS was used for the whole experimental period. Mycoplasma contamination was tested by PCR in the cultures and all of them were negative.

### Cell Characterization

Characterization of cultured cells was performed by immunofluorescence for fibroblasts (vimentin) and epithelial cells (cytokeratins) on CultureSlides® incubated with serum-free media (SFM) for 24 h. Immunofluorescence assessment was performed as previously reported [11]. The primary antibodies were against vimentin at dilution 1∶100 (V5255, Sigma, Saint Louis, Missouri, USA) and pan-cytokeratin at 1∶200 (C2562, recognizing cytokeratins 1, 4, 5, 6, 8, 10, 13, 18 and 19, Sigma). The percentage of positive cells was quantified using fluorescence microscopy.

### Study Design

When cultures placed in 150 cm^2^ flasks reached 80% confluence, FBS-supplemented media were switched to serum-free media (SFM) for 24 h. To analyze COX expression, the dynamics of MAPK phosphorylated forms and transcription factor nuclear translocation, cells were incubated with SFM in the presence or absence of IL-1β (R&D Systems Minneapolis, MN, USA) at different concentrations and for different times, depending on the protein analyzed. Meanwhile, in experiments on MAPK inhibition fibroblasts were pre-treated with ERK (PD98059), JNK (SP600125) and p38 MAPK (SB203580) inhibitors from Calbiochem (La Jolla, CL, USA) at different concentrations (0.1–10 µM) for 1 h, prior to the addition of 1 ng/ml IL-1β for 24 h. Total proteins were obtained by scraping the flasks after two washes with cold PBS. The cells were centrifuged (400 g, 5 min at 4°C) and then resuspended in different buffers, depending on the protein quantified and the analytical method.

### Analysis of COX Expression by Western Blot

Fibroblast cultures were incubated with IL-1β (1 ng/ml) from 0 to 24 h in time-course experiments, and for 24 h with IL-1β (0–10 ng/ml) in dose-response experiments. Cell pellet was resuspended in 0.4 ml ice-cold lysis buffer (Complete™ protease inhibitor cocktail tablet in 50 ml of 0.05 M Hepes buffer solution, 0.05% v/v Triton X-100, and 625 µM PMSF). Cells were sonicated twice for 15 sec in a sonifier (Branson, Danbury, CT, USA) and centrifuged (12,000 g, 10 min at 4°C). Supernatant containing total proteins was quantified by Lowry’s method and used to analyze COX-1 and COX-2 protein expression by Western blot, as described previously (11). The primary antibodies used were against COX-1 (SC-1752, Santa Cruz) or COX-2 (SC-1745, Santa Cruz) at dilution 1∶1000. Immunoreactive bands were visualized using a chemoluminiscent method (Supersignal West Pico Chemiluminescent Substrate, Rockford, IL, USA). Light emissions were detected by the CCD Camera System LAS 3000 (Fujifilm, Tokyo, Japan). Band intensities were quantified with Fujifilm Image Gauge 4.0 Software, and normalized by β-actin band intensities assessed in the same samples.

### Analysis of the Dynamics of MAPK Phosphorylated Forms by Western Blot

Fibroblast cultures were incubated with 1 ng/ml IL-1β from 0 to 60 min. Cell pellet was resuspended with an insulin syringe in ice-cold Rippa buffer (TrisHcl 50 mM+Nacl 150 mM, ph 7.4, apropine 1∶1000, leupeptine 1∶1000, ortovanadate 1∶1000, NaF 1 mM, DTT 1 mM, pefabloc 100 mg/ml, igepal 1%, SDS 0.1% and Na deoxicolat 0.5%). The samples were in ice for 1 h and then centrifuged (12,000 g, 10 min at 4°C). Supernatant containing total proteins was quantified by Lowry’s method and used to analyze phosphorylated and non-phosphorylated forms of MAPKs by Western blot. Briefly, 20 µg of proteins in loading buffer were denaturalized in a thermocycler (70°C, 10 min), loaded in 12% TRIS-glycine gels and ran (125 V, 90 min) in a Novex XCell ΙΙ Mini-Cell. Proteins were transferred (20 V, 2 h) to a 0.45 µm pore size nitrocellulose membrane and non-specific binding sites were blocked using blocking buffer (5% BSA, 0.1% Tween 20, in 10 nM TBS) for 1 h at room temperature in an orbital shaker. The membranes were then washed three times in 0.1% Tween 20, in 10 nM TBS and incubated overnight with the primary antibody buffer (5% BSA and 0.1% Tween 20 in 10 nM TBS). The primary antibodies used were p-p38 MAPK at 1∶1000 (9215, Cell Signalling Technology, Inc, Beberly, Mass, USA), p38 MAPK at 1∶1000 (9212, Cell Signalling), p-JNK at 1∶1000 (4668, Cell Signalling), JNK at 1∶1000 (9258, Cell Signalling), p-ERK at 1∶2000 (4370, Cell Signalling) and ERK at 1∶2000 (4695, Cell Signalling). The membranes were then washed four times in washing buffer (0.1% Tween 20 in 10 nM TBS) and incubated with peroxidase-conjugated secondary antibody (1∶3000) diluted in blocking buffer. After four washes, immunoreactive bands were visualized using a chemoluminiscent method (Supersignal West Dura Chemiluminescent Substrate, Rockford, IL, USA). Light emissions were detected by the CCD Camera System LAS 3000 (Fujifilm, Tokyo, Japan). Band intensities were quantified with Fujifilm Image Gauge 4.0 Software and normalized by the non-phosphorylated form of the respective MAPKs studied. The β-actin protein was assessed as a loading control.

### MAPK Inhibition Analysis by Western Blot

Cell pellet was resuspended in 0.4 ml ice-cold lysis buffer, sonicated twice for 15 sec and centrifuged (12,000 g, 10 min at 4°C). Supernatant containing total proteins was quantified by Lowry’s method and used to analyze COX-2 protein expression by Western blot, as described above.

### Isolation of Nuclear Proteins

Fibroblasts cultures were incubated with 10 ng/ml IL-1β from 0 to 60 min. Since activated transcription factors translocate to the nucleus, the Active Motif Nuclear Extract Protocol (Active Motif, Carlsbad, CA, USA) was used to isolate nuclear proteins. The purity of the nuclear fractions was assayed using LDH (AB 1222, Millipore, Chemicon International, Temecula, CA, USA) as a cytosolic marker.

### Analysis of Transcription Factors Translocation by ELISA-based Kits

The presence in nuclear extracts of p50, p52, p65, c/EBPα and C/EBPβ was measured with the ELISA-based kit TransAM® (Active Motif, Carlsbad, CA, USA), according to the manufacturer instructions. The colorimetric reading at 450 nm was determined in a microplate reader MultiScan Ascent (Thermo, Rockford, IL, USA). The positive-control Jurkat nuclear extract provided with the kit was used to quantify the samples and assess assay specificity.

### Analysis of Transcription Factor Translocation by Western Blot

Since Western blot was performed to confirm the TransAM® findings, only the transcription factors that showed an increase in translocation with this ELISA-based method were analyzed. Nuclear extracts (30 µg) quantified by the Lowry method were denaturalized in thermocycler, loaded in 7% TRIS-acetate gel and run (125 V for 90 min) in a Novex XCell ΙΙ Mini-Cell. The protein was then transferred (20 V 2 h) to an 0.45 µm pore-size nitrocellulose membrane and non-specific sites were blocked with blocking buffer for 1 h at RT in an orbital shaker. The membranes were incubated with the primary antibody against p65 (SC-372-G, Santa Cruz), p50 (SC-1190, Santa Cruz) in blocking buffer (1∶1,000). The membranes were then washed 4 times in 0.5 Tween 20 in 10 nmol/L PBS and incubated with the peroxidase-conjugated secondary antibody (1∶1000) diluted in blocking buffer. The bands were visualized as described in the COX Western blot section.

### Statistic Analysis

The data obtained from MAPK phosphorylation are expressed as median of the ratio phosphorylated:non-phosphorylated form. Results obtained from MPAK inhibition are expressed as medians and 25^th^–75^th^ interquartiles of the COX-2 expression, compared to IL-1β treated cells. Data obtained from ELISA-based kits are expressed as fold change increase median of the ratio transcription factor versus positive control. The non-parametric statistical Mann-Whitney U-test was used for between-group comparisons and the Wilcoxon test was used for paired comparisons. Statistical significance was set at *p*<0.05.

## Results

### COX Expression

**Figure 1:**
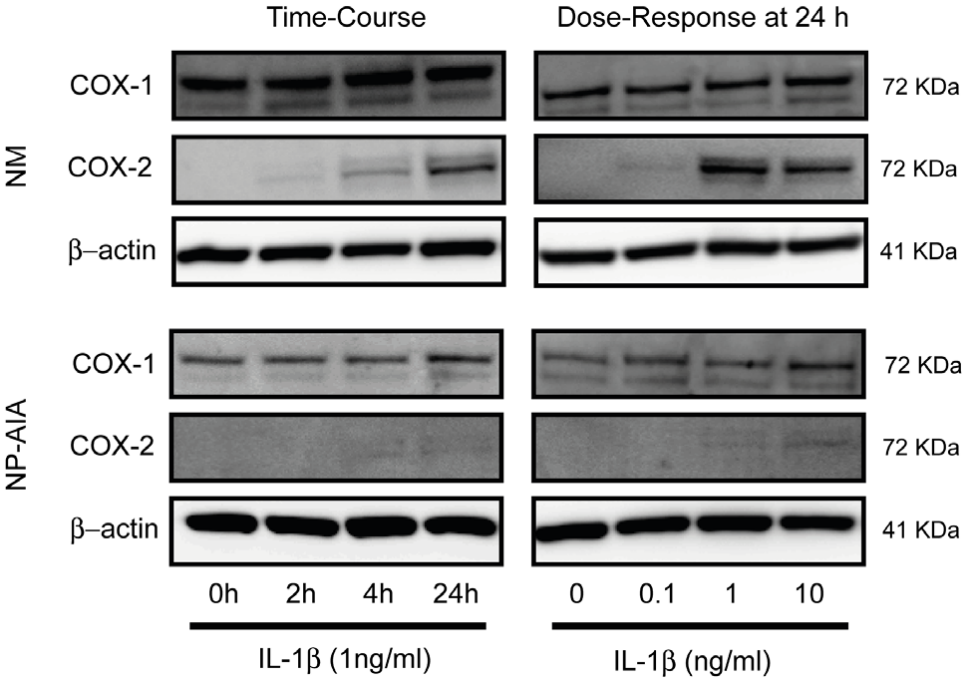
Time-course and dose-response of IL-1β in COX-1 and COX-2 protein expression. Fibroblasts from nasal mucosa (NM) and nasal polyps from AIA patients (NP-AIA) were incubated at different times and concentrations with IL-1β. COX-1 expression was not altered by IL-1β treatment in either NM or NP-AIA fibroblasts. In NM fibroblasts, COX-2 expression was induced in a time-dependent and dose-response manner. In NP-AIA, COX-2 protein expression was not significantly increased, compared to baseline level. The image that is shown is representative of NM and NP-AIA Western blot.

Basal COX-1 expression was not different between NM and NP-AIA fibroblasts. After IL-1β incubation in time-course and dose-response experiments, COX-1 expression was not modified in NM (N = 3) and NP-AIA (N = 3) cultured fibroblasts (**Figure 1**). COX-2, basal protein expression was not detected in either the NM or NP-AIA fibroblasts. However, fibroblasts from NM showed an increase in COX-2 expression, in a time-course and dose-response manner, after IL-1β incubation, with the highest COX-2 expression coming after 24 h of treatment with IL-1β at 1 and 10 ng/ml. In contrast, incubation with IL-1β at 24 h did not change COX-2 protein expression in cultured fibroblasts from the NP of AIA, compared to the baseline (**Figure 1**).

### MAPK Activation

**Figure 2:**
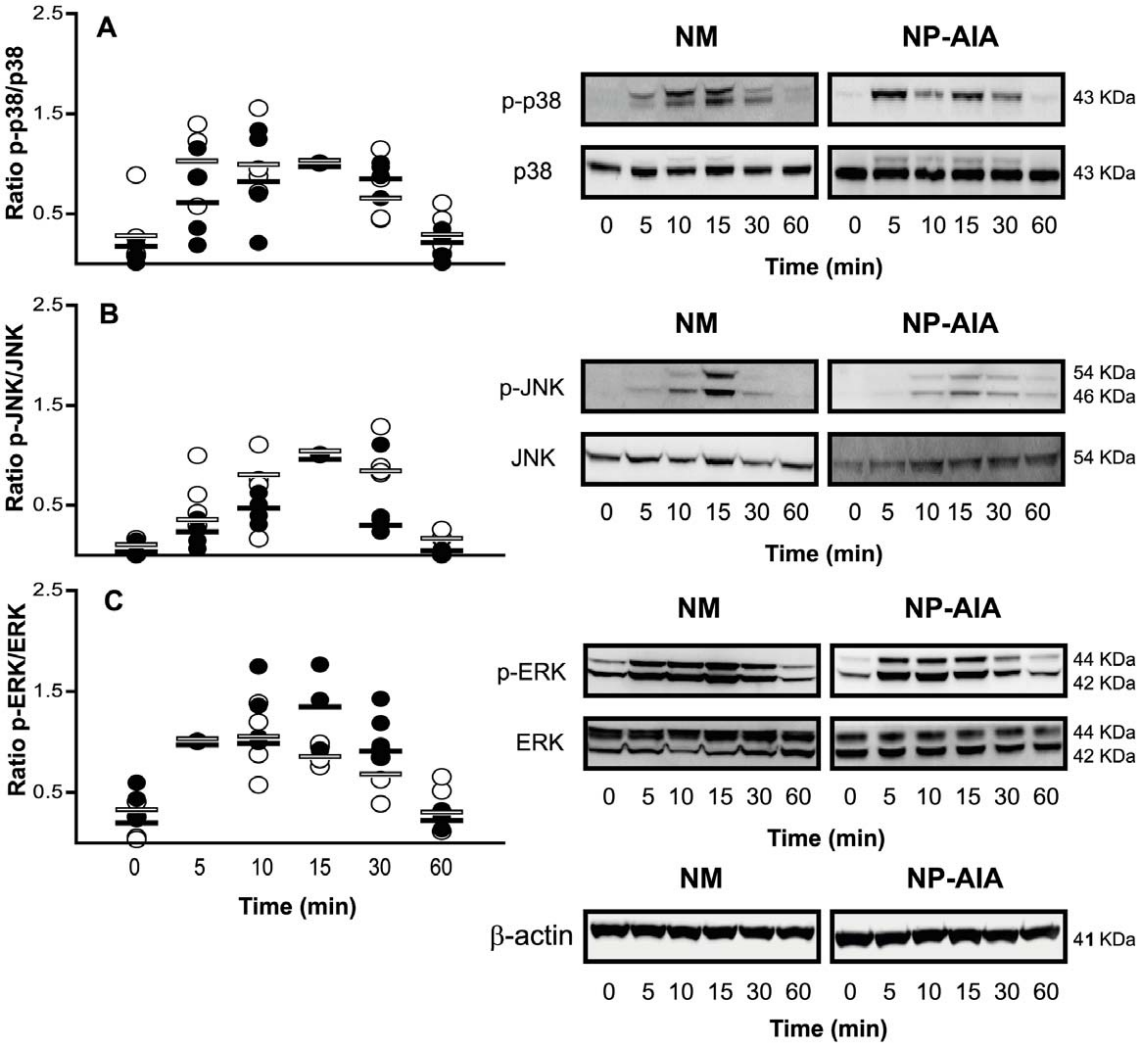
Time-course of MAPK activation by IL-1β in nasal fibroblasts cultures. Fibroblasts from nasal mucosa (NM, N = 4, black spots) and nasal polyps from AIA patients (NP-AIA, N = 4, white spots) were incubated with IL-1β (1 ng/ml) for 5 to 60 min. Phosphorylated and non-phosphorylated forms of p38 MAPK (A), JNK (B), and ERK (C) were measured by Western blot. Results are expressed as the ratio of phosphorylated *versus* non-phosphorylated MAPK forms. Graph shows individual experimental results and lines indicate the medians values. MAPK dynamic activation was not different (NS, Mann-Whitney U-test) between NM and NP-AIA fibroblasts. Insets show representative Western blot images of p38 MAPK, JNK, and ERK phosphorylation dynamics and β-actin as loading control.

A trend towards increased ratios of phosphorylated versus non-phosphorylated forms of the studied MAPKs (p38 MAPK, JNK and ERK) were observed after 5 min of IL-1β incubation in both NM and NP-AIA fibroblast cultures. In NM, the phosphorylation kinetics of p38 MAPK, JNK and ERK reached their maximum at 15 min. However, in NP-AIA the maximum phosphorylation level was observed at 5 min for both p38 MAPK and ERK, and at 15 min for JNK. The phosphorylation level of p38 MAPK, JNK and ERK reverted to close to the basal level at 60 min in both the NM and NP-AIA fibroblast cultures. Nevertheless the observed trends did not achieve statistical significance compared to baseline (**Figure 2**). A comparison of the ratios of phosphorylated versus non-phosphorylated MAPK forms showed no significant differences between NM and NP-AIA fibroblasts.

### MAPK Inhibition

**Figure 3:**
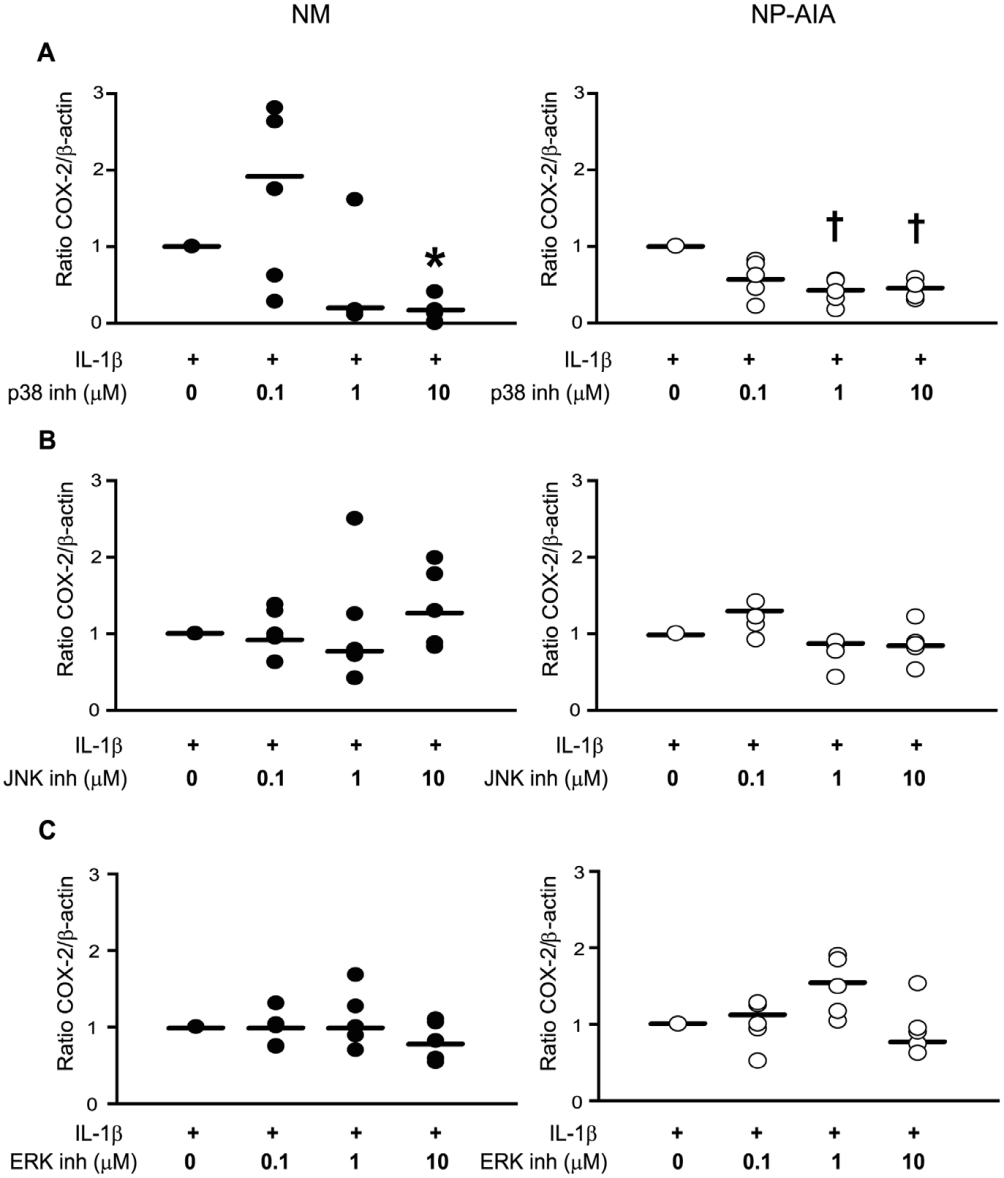
Effect of MAPK specific inhibitors on COX-2 protein expression. Fibroblasts from nasal mucosa (NM, N = 5, black spots) and nasal polyps from AIA patients (NP-AIA, N = 5, white spots) were pre-treated with p38 (A) MAPK (SB203580), (B) JNK (SP600125), and (C) ERK (PD98059) specific inhibitors at different concentrations (0.1–10 µM) for 1 h before addition of IL-1β (1 ng/ml) for 24 h. COX-2 and β-actin protein expression was analyzed by Western blot. Only the p38 MAPK inhibitor significantly blocked IL-1β-induced COX-2 expression in NM fibroblasts. Results are presented as COX-2/β-actin ratio. Graph shows individual experimental results and lines indicate the medians values. * p<0.05, † p = 0.06 compared to IL-1β treatment by Wilcoxon test.

In NM, incubation with p38 MAPK inhibitor SB203580 at 10 µM significantly decreased IL-1β-induced COX-2 protein expression (85%). In NP-AIA, p38 MAPK specific inhibitor decreased IL-1β-induced COX-2 protein expression at 1 µM (65%) and 10 µM (65%), although statistical significance was not achieved (*p* = 0.06), probably due to the low COX-2 induction levels in these cells. As regards JNK and ERK inhibition, both failed to modify COX-2 protein expression in NM and NP-AIA fibroblasts (**Figure 3**). Finally, when comparing the effects of the different specific inhibitors on COX-2 expression, no significant differences were found between NM and NP-AIA fibroblasts.

### Transcription Factor Nuclear Translocation

**Figure 4:**
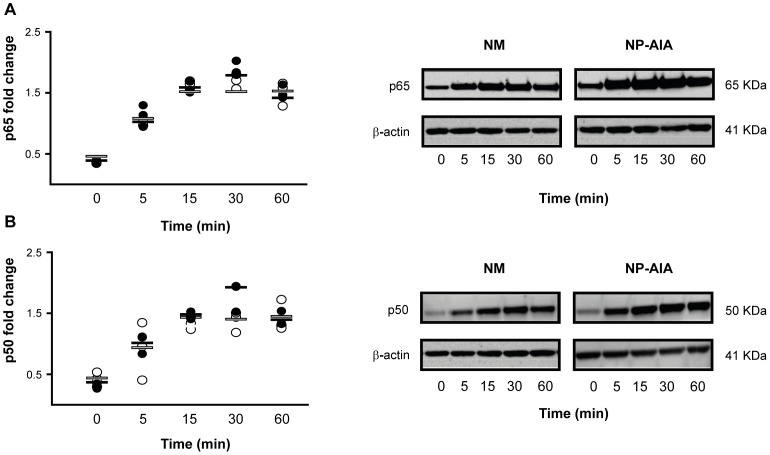
Time-course of p65 and p50 nuclear translocation induced by IL-1β in nasal fibroblast cultures. Fibroblasts from nasal mucosa (NM, N = 3, black spots) and nasal polyps from AIA patients (NP-AIA, N = 3, white spots) were incubated with IL-1β (10 ng/ml) for 5 to 60 min. p65 (A) and p50 (B) nuclear translocation were measured by TransAM® and Western blot. Graphs show the fold change increase from individual experimental results and lines indicate the medians values. Insets show representative Western blot images of p65 (A) and p50 (B) subunits from both NM and NP-AIA fibroblasts and the loading control β-actin. No significant differences (NS by Mann-Whitney U-test) were found at any time between NM and NP-AIA fibroblasts.

The analysis by TransAM® of p50 and p65 subunits showed the presence of these subunits at basal level in both NM and NP-AIA fibroblasts. Treatment with IL-1β induced the nuclear translocation of subunits p50 and p65, with a trend towards maximum translocation at 30 min and a decrease at 60 min in NM fibroblasts. In NP-AIA the trend towards maximum translocation was reached at 15 min and a plateau effect was maintained until 60 min. However, the observed trends did not achieve statistical significance compared to baseline. Western blot was performed with the same samples and the obtained images suggested similar kinetics **(** **Figure 4** **)**. TransAM® measures revealed that the nuclear presence of C/EBPα, C/EBPβ **(Figure S1)** and p52 was not modified after IL-1β incubation of NM and NP-AIA fibroblasts. No statistically significant differences were found compared at any time in p50, p65, p52, C/EBPα and C/EBPβ protein expression between NM and NP-AIA fibroblasts.

## Discussion

Reduced COX-2 expression, and consequently decreased PGE_2_ concentration, has previously been reported in peripheral blood leukocytes (PBLs) [27], urine [28], nasal polyps [29] and nasal polyp fibroblasts [7] in a variety of cells and samples from AIA patients, although the mechanism involved in this alteration remain unclear. To investigate the potential mechanisms underlying differences in the COX pathway of arachidonic acid in AIA, we used a previously published *in vitro* model [7] in which IL-1β induced lower COX-2 expression in NP-AIA fibroblasts compared with NM fibroblasts.

We hypothesized that the observed anomalies of COX-2 expression under inflammatory conditions were caused by differences in the signalling pathways, MAPK activation or nuclear transcription factor translocation between fibroblasts derived from NM and those obtained from NP of AIA patients.

The transient increase in phosphorylated levels in NP-AIA, peaking at 5 min (p38 MAPK and ERK) and 15 min (JNK) post-stimulation, were similar to those previously reported in stromal cells [30] and keratocyst fibroblasts [31], demonstrating the ability of IL-1β to activate MAPK signalling. To examine the involvement of the three main MAPK pathways in COX-2 gene expression regulation, NM and NP-AIA fibroblasts were incubated with selective MAPK inhibitors. ERK and JNK specific inhibitors had no significant effect on COX-2 induced expression. In contrast, a selective p38 MAPK inhibitor significantly reduced the IL-1β-induced COX-2 expression in NM fibroblasts, while only a tendency towards the inhibition of COX-2 expression was observed in NP. The latter finding can be explained by the difficulties in demonstrating differences in the inhibition of the expression of a gene (COX-2 gene) that changes very little after stimulation. All in all, these findings suggest that the p38 MAPK pathway is the transcription factor most involved in the regulation of COX-2 expression in nasal fibroblasts.

Our finding concurs with Chen *et al.* [32], who demonstrated the importance of p38 MAPK in COX-2 regulation in human pulmonary epithelial cells and the lack of involvement of ERK. Similarly, Ulivi *et al.* [21] also showed that p38 MAPK plays a critical role in COX-2 regulation in chondrocytes under inflammatory conditions.

IL-1β treatment induced rapid nuclear translocation of p50 and p65 NF-κB subunits in both NM and NP-AIA fibroblasts. In keeping with this result, previous reports have shown similar findings in several cell types, such as ASM cells [33], HT-29 [34] and lung fibroblasts [35], demonstrating p50 and p65 NF-κB involvement in gene expression regulation under inflammatory conditions, such as COX-2 induction. There were no statistical differences in translocation dynamics and nuclear concentrations of p65 and p50 proteins between NM and NP-AIA fibroblasts, suggesting that the nuclear translocation of NF-κB transcription factors is not altered in NP-AIA fibroblasts. In contrast, Picado *et al.* [36] reported a reduced NF-κB activity in NP-AIA samples when measuring p65 and p50 subunits. These discrepancies could be explained by the samples used (whole NP tissue explants), the experimental design followed (unstimulated samples) and the heterogeneity of NP-AIA tissues (some of the patients were receiving nasal corticosteroids before surgery).

In our study, the results obtained for the NF-κB subunit p52 did not show any variation after IL-1β induction, suggesting that p52 is not activated under the experimental conditions used in the study. Similarly, C/EBP isoform assessment revealed no changes after IL-1β incubation, suggesting that C/EBPs are not activated through the IL-1β pathway in either NM or NP-AIA fibroblasts. C/EBP involvement in COX-2 regulation has been demonstrated in several cell models, such as macrophages [37] and human foreskin fibroblasts [38]. However, the stimuli used in these studies were endotoxin and PMA, respectively, and this difference may account for the C/EBP activation found in these experimental models.

We could not find any differences in the nuclear translocation of p65, p50 and C/EBP isoforms in NP-AIA fibroblasts compared to NM fibroblasts after IL-1β induction. This observation concurs with the findings of Coward *et al.* [35], who demonstrated that the expression of p65 and C/EBPβ was unaltered in fibroblasts from idiopathic pulmonary fibrosis patients compared to control fibroblasts, a disease that is also characterized by down-regulated COX-2 expression in fibroblasts.

In summary, our study demonstrates that only p38 MAPK plays a role in COX-2 induction by IL-1β. Moreover, there were similar nuclear translocation dynamics in the NF-κB subunits, and no differences in C/EBP regulation were found in fibroblasts from either NP-AIA or NM. In conclusion, in the present study we did not find any alterations responsible for the COX-2 down-regulation described in NP-AIA fibroblasts.

Finally, future studies should assess other mechanisms, such as transcription factors binding ability to gene promoter, the activity of histone acetyltransferases and deacetylases and mRNA stability, which could be crucial to understanding the observed down-regulation of COX-2 in aspirin-induced asthma.

## Supporting Information

Figure S1 Time-course of c/EBPα and c/EBPβ nuclear translocation induced by IL-1β in nasal fibroblast cultures. Fibroblasts from nasal mucosa (NM, N = 3, black spots) and nasal polyps from AIA patients (NP-AIA, N = 3, white spots) were incubated with IL-1β (10 ng/ml) for 5 to 60 min. c/EBPα (A) and c/EBPβ (B) nuclear translocation were measured by TransAM®. Graphs show the fold change increase from individual experimental measures and the medians. No significant differences (NS by Mann-Whitney U-test) were found at any time between NM and NP-AIA fibroblasts.
(TIF)

## References

[1] Stevenson DD, Szczeklik A (2006) Clinical and pathologic perspectives on aspirin sensitivity and asthma. J Allergy Clin Immunol 118: 773–86. 17030227 10.1016/j.jaci.2006.07.024

[2] Fokkens WJ, Lund VJ, Mullol J, Bachert C, Alobid I, et al (2012) EPOS 2012: European position paper on rhinosinusitis and nasal polyps 2012. A summary for otorhinolaryngologists. Rhinology 50: 1–12. 22469599 10.4193/Rhino12.000

[3] Picado C (2006) Mechanisms of aspirin sensitivity. Curr Allegy Asthma Rep 6: 198–202. 10.1007/s11882-006-0035-2 16579869

[4] Kowalski ML, Pawliczak R, Wozniak J, Siuda K, Poniatowska M, et al (2000) Differential metabolism of arachidonic acid in nasal polyp epithelial cells cultured from aspirin-sensitive and aspirin-tolerant patients. Am J Respir Crit Care Med 16: 391–8. 10.1164/ajrccm.161.2.9902034 10673176

[5] Schäfer D, Schmid M, Göde UC, Baenkler HW (1999) Dynamics of eicosanoids in peripheral blood cells during bronchial provocation in aspirin-intolerant asthmatics. Eur Respir J 13: 638–46. 10232440 10.1183/09031936.99.13363899

[6] Pierzchalska M, Szabó Z, Sanak M, Soja J, Szczeklik A (2003) Deficient prostaglandin E2 production by bronchial fibroblasts of asthmatic patients, with special reference to aspirin-induced asthma. J Allergy Clin Immunol 111: 1041–8. 12743569 10.1067/mai.2003.1491

[7] Roca-Ferrer J, Garcia-Garcia FJ, Pereda J, Perez-Gonzalez M, Pujols L, et al (2011) Reduced expression of COXs and production of prostaglandin E(2) in patients with nasal polyps with or without aspirin-intolerant asthma. J Allergy Clin Immunol 128: 66–72. 21397936 10.1016/j.jaci.2011.01.065

[8] Simmons DL, Botting RM, Hla T (2004) Cyclooxygenase isozymes: the biology of prostaglandin synthesis and inhibition. Pharmacol Rev 56: 387–437. 15317910 10.1124/pr.56.3.3

[9] Roca-Ferrer J, Pujols L, Gartner S, Moreno A, Pumarola F, et al (2006) Upregulation of COX-1 and COX-2 in nasal polyps in cystic fibrosis. Thorax 61: 592–6. 16517580 10.1136/thx.2004.039842 PMC2104672

[10] Xaubet A, Roca-Ferrer J, Pujols L, Ramírez J, Mullol J, et al (2004) Cyclooxygenase-2 is up-regulated in lung parenchyma of chronic obstructive pulmonary disease and down-regulated in idiopathic pulmonary fibrosis. Sarcoidosis Vasc Diffuse Lung Dis 21: 35–42. 15127973

[11] Dong C, Davis RJ, Flavell RA (2002) Map kinases in the immune response. Ann Review Immunol 20: 55–72. 10.1146/annurev.immunol.20.091301.131133 11861597

[12] Raman M, Chen W, Cobb MH (2000) Differential regulation and properties of MAPKs. Oncogene 26: 3100–12. 10.1038/sj.onc.1210392 17496909

[13] Nieminen R, Leinonen S, Lahti A, Vuolteenaho K, Jalonen U, et al (2005) Inhibitors of mitogen-activated protein kinases downregulate COX-2 expression in human chondrocytes. Mediators Inflamm 5: 249–55. 10.1155/MI.2005.249 PMC1279039 16258191

[14] Caughey GE, Cleland LG, Penglis PS, Gamble JR, James MJ (2001) Roles of Cyclooxygenase (COX)-1 and COX-2 in Prostanoid Production by Human Endothelial Cells: Selective Up-Regulation of Prostacyclin Synthesis by COX-2. Journal Immunol 167: 2831–8. 11509629 10.4049/jimmunol.167.5.2831

[15] Laporte JD, Moore PE, Lahiri T, Schwartzman IN, Panettieri RA Jr, et al (2000) p38 MAP kinase regulates IL-1 beta responses in cultured airway smooth muscle cells. Am J Physiol Lung Cell Mol Physiol 279: L932–41. 11053030 10.1152/ajplung.2000.279.5.L932

[16] Chun K-S, Surh Y-J (2004) Signal transduction pathways regulating cyclooxygenase-2 expression: potential molecular targets for chemoprevention. Biochem Pharmacol 68: 1089–100. 15313405 10.1016/j.bcp.2004.05.031

[17] Ackerman WE 4th, Summerfield TLS, Vandre DD, Robinson JM, Kniss DA (2008) Nuclear factor-kappa B regulates inducible prostaglandin E synthase expression in human amnion mesenchymal cells. Biol Reprod 78: 68–76. 17928629 10.1095/biolreprod.107.061663

[18] Griffin BD, Moynagh PN (2006) Persistent interleukin-1beta signaling causes long term activation of NFkappaB in a promoter-specific manner in human glial cells. J Biol Chem 281: 10316–26. 16455661 10.1074/jbc.M509973200

[19] Branski RC, Zhou H, Sandulache VC, Chen J, Felsen D, et al (2010) Cyclooxygenase-2 signaling in vocal fold fibroblasts. Laryngoscope 120: 1826–31. 20717945 10.1002/lary.21017 PMC3132797

[20] Syeda F, Grosjean J, Houliston RA, Keogh RJ, Carter TD, et al (2006) Cyclooxygenase-2 induction and prostacyclin release by protease-activated receptors in endothelial cells require cooperation between mitogen-activated protein kinase and NF-kappaB pathways. J Biol Chem 281: 11792–804. 16467309 10.1074/jbc.M509292200

[21] Ulivi V, Giannoni P, Gentili C, Cancedda R, Descalzi F (2008) p38/NF-kB-dependent expression of COX-2 during differentiation and inflammatory response of chondrocytes. J Cell Biochem 104: 1393–406. 18286508 10.1002/jcb.21717

[22] Birkenkamp KU, Tuyt LM, Lummen C, Wierenga AT, Kruijer W, et al (2000) The p38 MAP kinase inhibitor SB203580 enhances nuclear factor-kappa B transcriptional activity by a non-specific effect upon the ERK pathway. Br J Pharmacol 131: 99–107. 10960075 10.1038/sj.bjp.0703534 PMC1572293

[23] Wu KK, Liou J-Y, Cieslik K (2005) Transcriptional Control of COX-2 via C/EBPbeta. Arterioscler Thromb Vasc Biol 25: 679–85. 15681294 10.1161/01.ATV.0000157899.35660.61

[24] Joo M, Park GY, Wright JG, Blackwell TS, Atchison ML, et al (2004) Transcriptional regulation of the cyclooxygenase-2 gene in macrophages by PU.1. J Biol Chem 279: 6658–65. 14966110 10.1074/jbc.M306267200

[25] Cho YH, Lee CH, Kim SG (2003) Potentiation of Lipopolysaccharide-Inducible Cyclooxygenase 2 Expression by C2-Ceramide via c-Jun N-Terminal Kinase-Mediated Activation of CCAAT/Enhancer Binding Protein β in Macrophages. Molecular Pharmacol 63: 512–23. 10.1124/mol.63.3.512 12606757

[26] Casadevall J, Ventura PJ, Mullol J, Picado C (2000) Intranasal challenge with aspirin in the diagnosis of aspirin intolerant asthma: evaluation of nasal response by acoustic rhinometry. Thorax 55: 921–4. 11050260 10.1136/thorax.55.11.921 PMC1745635

[27] Jedrzejczak-Czechowicz M, Lewandowska-Polak A, Bienkiewicz B, Kowalski ML (2008) Involvement of 15-lipoxygenase and prostaglandin EP receptors in aspirin-triggered 15-hydroxyeicosatetraenoic acid generation in aspirin-sensitive asthmatics. Clin Exp Allergy 38: 1108–16. 18462455 10.1111/j.1365-2222.2008.02989.x

[28] Higashi N, Mita H, Ono E, Fukutomi Y, Yamaguchi H, et al (2010) Profile of eicosanoid generation in aspirin-intolerant asthma and anaphylaxis assessed by new biomarkers. J Allergy Clin Immunol 125: 1084–1091.e6. 20304469 10.1016/j.jaci.2009.12.977

[29] Pujols L, Mullol J. Alobid I, Roca-Ferrer J, Xaubet A, et al (2004) Dynamics of COX-2 in nasal mucosa and nasal polyps from aspirin-tolerant and aspirin-sensitive asthmatics. J Allergy Clin Immunol 114: 814–9. 15480320 10.1016/j.jaci.2004.07.050

[30] Wu MH, Wang CA, Lin CC, Chen LC, Chang WC, et al (2005) Distinct regulation of cyclooxygenase-2 by interleukin-1beta in normal and endometriotic stromal cells. J Clin Endocrinol Metab 90: 286–95. 15483103 10.1210/jc.2004-1612

[31] Ogata S, Kubota Y, Yamashiro T, Takeuchi H, Ninomiya T, et al (2007) Signaling pathways regulating IL-1alpha-induced COX-2 expression. J Dent Res 86: 186–91. 17251521 10.1177/154405910708600215

[32] Chen P, Cai Y, Yang ZG, Zhou R, Zhang GS, et al (2006) Involvement of PKC, p38 MAPK and AP-2 in IL-1β eta-induced expression of cyclooxygenase-2 in human pulmonary epithelial cells. Respirology 11: 18–23. 16423197 10.1111/j.1440-1843.2006.00779.x

[33] Nie M, Pang L, Inoue H, Knox AJ (2003) Transcriptional regulation of cyclooxygenase 2 by bradykinin and interleukin-1beta in human airway smooth muscle cells: involvement of different promoter elements, transcription factors, and histone h4 acetylation. Mol Cell Biol 23: 9233–44. 14645533 10.1128/MCB.23.24.9233-9244.2003 PMC309638

[34] Liu W, Reinmuth N, Stoeltzing O, Parikh AA, Tellez C, et al (2003) Cyclooxygenase-2 is up-regulated by interleukin-1 beta in human colorectal cancer cells via multiple signaling pathways. Cancer Res 63: 3632–6. 12839952

[35] Coward WR, Watts K, Feghali-Bostwick CA, Knox A, Pang L (2009) Defective histone acetylation is responsible for the diminished expression of cyclooxygenase 2 in idiopathic pulmonary fibrosis. Mol Cell Biol 29: 4325–39. 19487460 10.1128/MCB.01776-08 PMC2715818

[36] Picado C, Bioque G, Roca-Ferrer J, Pujols L, Mullol J, et al (2003) Nuclear factor-kappaB activity is down-regulated in nasal polyps from aspirin-sensitive asthmatics. Allergy 58: 122–6. 12622742 10.1034/j.1398-9995.2003.23792.x

[37] Wadleigh DJ, Reddy ST, Kopp E, Ghosh S, Herschman HR (2000) Transcriptional activation of the cyclooxygenase-2 gene in endotoxin-treated RAW 264.7 macrophages. J Biol Chem 275: 6259–66. 10692422 10.1074/jbc.275.9.6259

[38] Saunders MA, Sansores-Garcia L, Gilroy DW, Wu KK (2001) Selective suppression of CCAAT/enhancer-binding protein beta binding and cyclooxygenase-2 promoter activity by sodium salicylate in quiescent human fibroblasts. J Biol Chem 276: 18897–904. 11278846 10.1074/jbc.M011147200

