# Supplementary figures and images for: Signal Transduction Pathways (MAPKs, NF-κB, and C/EBP) Regulating COX-2 Expression in Nasal Fibroblasts from Asthma Patients with Aspirin Intolerance
Source: PLoS One. 2012 Dec 11;7(12):e51281. doi: 10.1371/journal.pone.0051281 (PMC3519844; doi:10.1371/journal.pone.0051281)

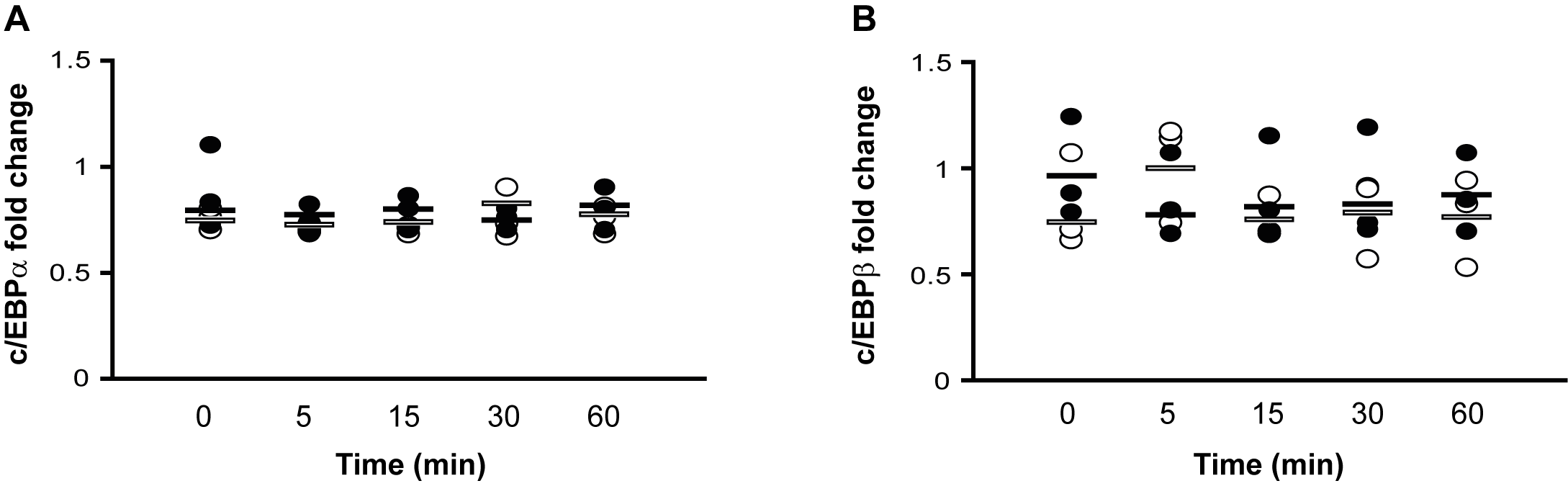

Supplement: Figure S1 — Time-course of c/EBPα and c/EBPβ nuclear translocation induced by IL-1β in nasal fibroblast cultures. Fibroblasts from nasal mucosa (NM, N = 3, black spots) and nasal polyps from AIA patients (NP-AIA, N = 3, white spots) were incubated with IL-1β (10 ng/ml) for 5 to 60 min. c/EBPα (A) and c/EBPβ (B) nuclear translocation were measured by TransAM®. Graphs show the fold change increase from individual experimental measures and the medians. No significant differences (NS by Mann-Whitney U-test) were found at any time between NM and NP-AIA fibroblasts. (TIF) [file pone.0051281.s001.tif]
